# Characteristics of energy metabolism and stress load in elite MOBA E-sports athletes

**DOI:** 10.3389/fphys.2026.1716237

**Published:** 2026-02-06

**Authors:** Ziyang Li, Mengli Wei, Yaping Zhong, Yiyuan Chong, Minghui Li

**Affiliations:** School of Sports Training, Wuhan Sports University, Wuhan, China

**Keywords:** cognitive load, energy metabolism, e-sports, heart rate variability, league of legends

## Abstract

**Background:**

E-sports gained growing recognition as a competitive pursuit with quantifiable physiological demands. While previous studies have shown that cognitive stress modulates energy metabolism and autonomic nervous system regulation, phase-specific physiological responses during gameplay remain poorly understood.

**Methods:**

20 elite League of Legends players (rank ≥ Platinum) were enrolled. Their cardiopulmonary function and autonomic nervous system activity were monitored during rest and across three distinct match phases (early-, mid-, and late-game). Energy metabolism parameters were measured using a portable cardiopulmonary testing system, and heart rate variability indices were assessed with a chest-worn monitor.

**Results:**

Compared with the resting state, the early-game elicited significant increases in energy expenditure oxygen consumption (VO_2_), carbon dioxide production (VCO_2_), and respiratory exchange ratio (all p < 0.001), with carbohydrate oxidation accounting for approximately 63% of total energy supply. Heart rate (HR) increased by 12.8%, while the root mean square of successive differences (RMSSD) rose by 52.2%, indicating sympathetic-parasympathetic coactivation. In the mid-game, metabolic indices declined but remained above baseline levels, characterized by sustained carbohydrate dominance (about 63.6%) and increased fat oxidation (about 30.2%); heart rate variability indices reflected sympathetic predominance accompanied by partial parasympathetic recovery. The late-game was characterized by slight rebounds in metabolic load and carbohydrate utilization (about 68.2%), accompanied by decreased Heart rate and elevated RMSSD, suggesting partial autonomic recovery alongside incipient neural fatigue.

**Conclusion:**

Elite e-sports athletes demonstrate dynamic, phase-dependent alterations in energy metabolism and autonomic nervous system regulation. The early phase is characterized by carbohydrate-dominated physiological activation, the mid phase by metabolic stabilization amid sustained cognitive demand, and the late phase by partial autonomic recovery with cumulative neural fatigue. These findings highlight the physiological mechanisms underlying E-sports performance and provide actionable insights for optimizing training regimens, fatigue monitoring protocols, and recovery interventions.

## Highlights

- For the first time, this study systematically analyzed the dynamic differences in physiological energy metabolism and stress load of elite MOBA e-sports athletes across three distinct match phases: the early (0–10 min), middle (11–25 min), and late (≥26 min) phases of MOBA games, thereby addressing a key limitation of prior research that relied on homogenizing whole-game analysis.
- The core physiological rules of the three phases were clarified. During the early phase, energy metabolism increased sharply (with carbohydrate-dominated energy supply) and stress load peaked; in the middle phase, metabolic indices decreased but remained above baseline levels (with a higher proportion of fat oxidation contributing to energy supply), and sympathetic-parasympathetic regulation tended toward equilibrium; in the late phase, metabolism showed a slight rebound (with a further increased proportion of carbohydrate oxidation for energy supply), and athletes manifested cumulative neural fatigue. These results explicitly confirm the phase-specific physiological alterations underlying MOBA gameplay.
- Based on the phase characteristics, E-sports training schemes were proposed: enhancing energy mobilization capacity under cognitive stress during the early-game; utilizing heart rate variability (HRV) for fatigue monitoring throughout the mid- and late-game; and incorporating neural recovery interventions in the late-game. This achieves precise alignment between phase-specific physiological traits and targeted training/intervention approaches.

## 1 Introduction

On 11 February 2025, the International Olympic Committee (IOC) officially declared that the first “E-sports Olympic Games” would be held in Riyadh, Saudi Arabia, in 2027. This landmark event marks the first time that e-sports has been incorporated into the Olympic framework as an independent sport. E-sports is a competitive form highly dependent on information technology and mental confrontation; although it involves relatively little external physical activity, the challenges it poses to athletes’ cognitive, psychological, and physiological loads have attracted increasing attention (Jenny et al., 2016).

The essence of human movement lies in the synergy between movement and energy metabolism: its external manifestation is visible physical activity, while its internal mechanism relies on the continuous support of energy metabolism processes (Li et al., 2014). Relevant studies have shown that during e-sports competitions, athletes’ physiological indicators, such as respiratory rate, oxygen uptake, and carbon dioxide emission, increase significantly. This indicates that e-sports is not a sedentary behavior in the traditional sense, but a special form of exercise with a distinct energy metabolism load (Kocak, 2021). Furthermore, the characteristics of energy metabolism are also affected by multiple factors, including exercise intensity, duration, and task nature. Among these factors, studies have pointed out that under conditions of short-duration exercise with high cognitive load, carbohydrate metabolism becomes the dominant energy supply mode (Mul et al., 2015). This is particularly important for e-sports: as a competitive discipline characterized by sustained high concentration and rapid, complex motor operations, it constitutes a typical high-cognitive-demand, short-term explosive competitive activity. Energy substrate utilization in esports may shift from fat oxidation to carbohydrate oxidation to fulfill the central nervous system’s immediate requirement for rapid energy provision (Laborde et al., 2017). In addition, energy metabolism also exhibits a regular pattern of dynamic fluctuations across different exercise phases. Such metabolic characteristics not only affect competitive performance but also provide important references for the formulation of competitive strategies, fatigue management, and the arrangement of recovery mechanisms (Li, 2013).

A number of studies have hitherto been conducted, which have furnished preliminary insights into the physiological responses of participants during esports. For instance, Kocak et al. monitored respiratory-metabolic indices in amateur esports players under resting and gaming conditions, reporting that METs and EE were significantly higher during gameplay than at rest, overall corresponding to light-intensity physical activity (Kocak, 2021). Another study by Haupt et al. demonstrated that, although players exhibited responses such as increased heart rate during gameplay, changes in oxygen consumption and energy expenditure during gameplay were not significant (Haupt et al., 2021). In addition, Nicholson et al. observed significant increases in EE and respiratory gas exchange variables during competitive gameplay in comparison with rest periods, accompanied by a marked increase in HR and a shortening of the R-R interval. However, neither time-domain nor frequency-domain HRV indices exhibited significant changes (Nicholson et al., 2024). Although these studies have provided certain data support for the characteristics of energy metabolism and autonomic nervous responses in e-sports, they still have several limitations: (1) Based on the duration of MOBA competitions, a match can be divided into three distinct phases: early game, mid game, and late game. Athletes demonstrate unique physiological and psychological responses across these phases, thereby inducing fluctuations in energy metabolism. Therefore, it is imperative to conduct phase-specific observations of energy metabolism data and summarize the corresponding energy supply patterns. Yet, existing studies treat the entire match as a homogeneous unit and have not addressed phase-specific differences in athletes’ energy metabolism; (2) Additionally, the energy expenditure (EE) of esports athletes is strongly associated with autonomic nervous system (ANS) regulation induced by the stress load endured during matches. Thus, it is essential to combine data on athletes’ autonomic nervous activity to gain an in-depth understanding of the causes of fluctuations in their energy metabolism.

This study takes elite Multiplayer Online Battle Arena (MOBA) e-sports athletes in League of Legends as subjects. A portable cardiopulmonary testing system and heart rate variability (HRV) monitoring equipment were employed to systematically gather energy metabolism data and HRV indices during the early-game, mid-game, and late-game phases of real matches. The primary objective was to investigate the dynamic changes in energy metabolism characteristics and autonomic nervous system regulation among these athletes.

## 2 Methods

### 2.1 Subject selection

A total of 25 male subjects were recruited for this study. The inclusion criteria were as follows: (1)Being a player of the League of Legends game; (2)Having a rank no lower than Platinum in the game’s solo/duo queue system (Nicholson et al., 2024); (3)Being in good health, with no metabolic diseases or cardiovascular disorders (Nicholson et al., 2024). The exclusion criteria were as follows: (1) taking metabolism-influencing drugs or health supplements during the study period; (2) engaging in high-intensity exercise during the study period; and (3) failing to control diet and sleep in accordance with the experimental requirements. After screening, 20 subjects were ultimately enrolled. All participants were fully informed of the experimental procedures and potential adverse reactions, and provided written informed consent. The basic information of the subjects and their rank distribution are presented in Tables 1, 2. Participants’ demographic characteristics and rank distribution are summarized in Tables 1, 2. All experimental procedures were performed in accordance with the Declaration of Helsinki. The study protocol and related ethical considerations were approved by the Ethics Committee of Wuhan Sports University (Approval No.: 2025143).

**TABLE 1:** Basic characteristics of the subjects (N = 20).

Variables	Values (mean ± SD)
Age (years) Height (cm) Weight (kg)	24.35 ± 1.78 177.85 ± 5.79 73.30 ± 7.83
BMI (kg/m^2^)	23.25 ± 2.10
Gaming experience (years)	5.50 ± 1.95

Data are presented as mean ± SD. BMI, body mass index.

**TABLE 2:** Distribution of game ranks.

Rank tier	Number
Platinum (IV-I) Emerald (IV-I)	10 5
Diamond (IV-I) Master Grandmaster	2 2 1

Data are presented as frequency.

### 2.2 Experimental equipment

**FIGURE 1:**
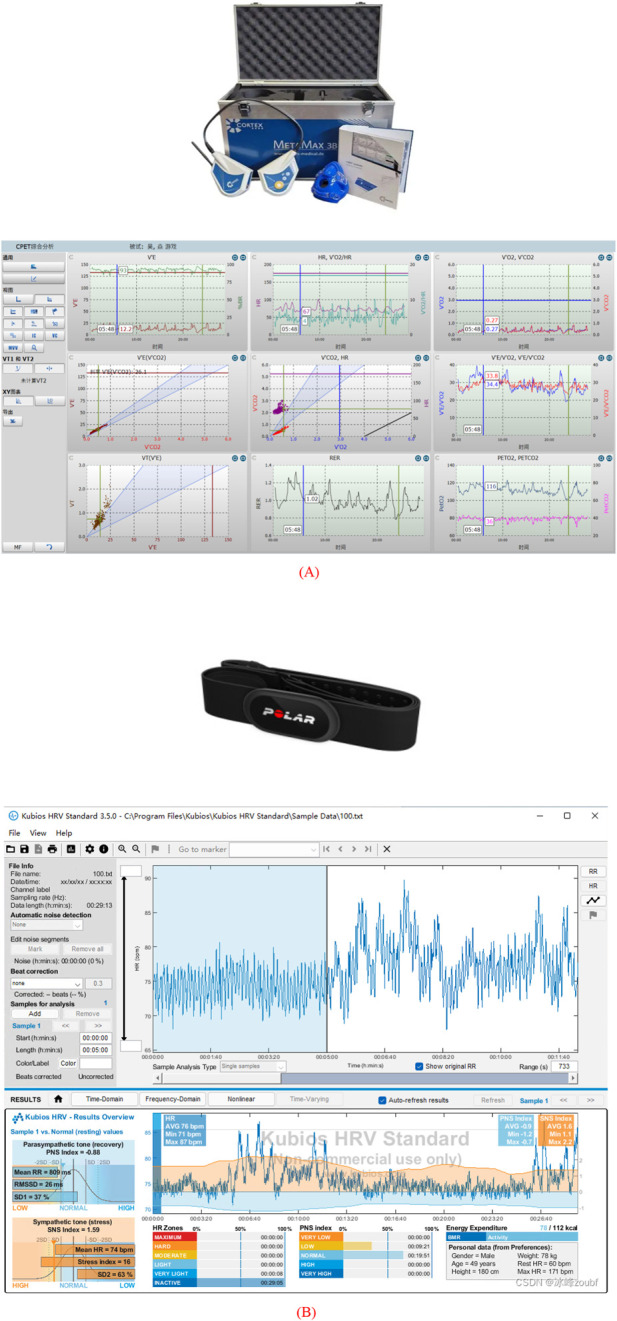
**(A)** Illustrates the MetaMax3B device and the collection diagram of metabolic gases; **(B)** Illustrates the Polar H10 heart rate strap and the monitoring diagram via Kubios software.

In this study, the MetaMax 3B portable cardiopulmonary function tester (manufactured by Cortex, Germany) was used for real-time collection of subjects’ energy metabolism parameters, and a chest-worn heart rate monitor (Polar H10) was paired with it for collecting HRV index data (Figure 1).

The cardiopulmonary function testing system uses an electrochemical sensor to monitor oxygen (O_2_) concentration and an infrared sensor to monitor carbon dioxide (CO_2_) concentration, with a sampling frequency of 10 Hz. The measurement accuracy is ±0.02vol% for O_2_ and ±0.01vol% for CO_2_; the flow measurement range is 0–250 L/min, and the measurement error is controlled within±2%, which can meet the requirements of physiological data collection under different exercise intensity conditions.

During the measurement, participants wore a customized mask and a head-mounted sampling device, with exhaled air directed to the main unit via a one-way valve system for analysis. Before the formal testing, a two-step calibration process was completed in compliance with the manufacturer’s operation manual: gas calibration (using a certified standard gas mixture) and volume calibration (via a 3-L standard syringe). Collected data were transmitted in real time to Cortex’s proprietary analysis software (MetaSoft Studio), enabling subsequent calculation and visual analysis of energy metabolism indices.

The heart rate monitor adopts electrocardiogram (ECG) technology, which measures heart rate by detecting the electrical activity generated during myocardial contraction. It is paired with the Kubios software (University of Eastern Finland) for HRV analysis. This software typically has a heart rate monitoring range of 30–240 bpm and a sampling rate of 1000 Hz, and can detect multiple HRV indicators such as HR and R-R interval.

Before the test, 75% alcohol wipes were used to clean the skin in the wearing area (to remove sweat and oil); subjects with thick chest hair were required to shave the local chest hair to ensure close contact between the electrode pads and the skin. During the test, the heart rate monitor was worn 2–3 cm below the midline of the sternum, with the sensor module aligned directly in front of the heart and wrapped horizontally around the chest. The tightness of the chest strap was adjusted to a level where “1-2 fingers could be inserted without obvious pressure”. After wearing, the Kubios software was used to observe the ECG waveform until it stabilized; if the waveform was disordered, the wearing position needed to be adjusted or the skin re-cleaned until the signal became stable.

### 2.3 Experimental protocol

**FIGURE 2:**
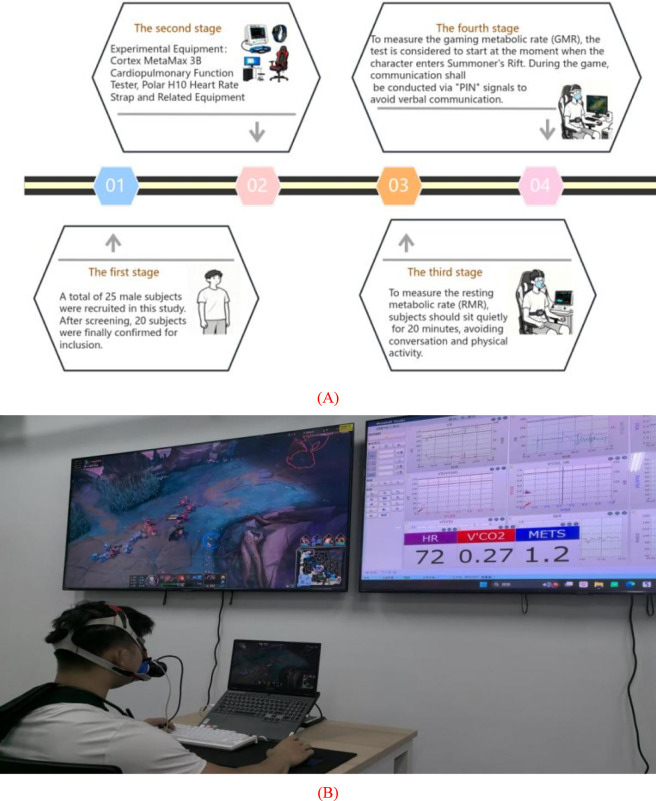
**(A)** Experimental flowchart; **(B)** Experimental setup.

To ensure the scientific validity and reproducibility of the experimental data, a series of pretest control conditions was established for the subjects (Figure 2). All subjects were required to meet the following requirements before the test:

1. Fasting for at least 3 h;
2. No alcohol consumption within 24 h prior to the experiment;
3. No smoking within 2 h before the start of the experiment;
4. Avoiding intake of any caffeinated foods or beverages (including but not limited to coffee, tea, energy drinks, etc.) within 8 h before the start of the experiment;
5. Avoiding moderate-to-high intensity physical activity on the day before the test and ensuring a sleep duration of no less than 7 h.

Before conducting the game task, the resting metabolic rate (RMR) of the subjects was first measured. Subjects were instructed to wear the MetaMax 3B cardiopulmonary function tester and the Polar H10 heart rate monitor, then sit quietly on an ergonomically designed e-sports chair for 20 min in a resting state. During the measurement, all conversations and physical activities were prohibited to minimize the impact of external interference on metabolic data. The experimental environment was controlled in a closed laboratory with a constant temperature (22 °C–24 °C), low illumination, and quiet conditions to ensure minimal external stimulation.

Immediately after the resting metabolic test, the subjects began the game-phase task. All subjects used the same model of computer, monitor, keyboard-mouse set, and headphones, and loaded into a solo-queued ranked game. The starting point of the game test was defined as the moment when the game character finished loading and started moving on the Summoner’s Rift map. To further minimize the confounding effects of linguistic and social variables on physiological indices, verbal communication and in-person interaction were prohibited during data collection; all communication was restricted to the game’s built-in signaling system (e.g., the “PIN” function).

### 2.4 Game phase division

Based on the average pace of professional leagues (LPL, LCK, LEC, and LCS) and the timing of key events, (Oracle’s Elixir, 2024; Riot Games, 2024) combined with empirical studies by scholars such as Gaina (2018) and Zhang and Naidu (2024), this study divided League of Legends matches into three phases: early game (0–10 min), mid game (11–25 min), and late game (26 min to the end of the game) (Gaina, 2018; Zhang and Naidu, 2024). This division ensures the scientific validity and standardization of subsequent analyses.

### 2.5 Indicator analysis

#### 2.5.1 Analysis of energy metabolism indicators

Energy metabolism indicators were collected using the Cortex cardiopulmonary function testing system, including oxygen consumption (VO_2_),carbon dioxide production (VCO_2_),minute ventilation (VE),respiratory exchange ratio (RER),metabolic equivalents (METs),and energy expenditure (EE). To ensure data stability and the accuracy of analysis results, when measuring RMR, only the physiological indicator signals from the middle 10 consecutive minutes were retained, while the data from the first 5 min and last 5 min were excluded to avoid interference from adaptation and recovery processes. During the game task phase, data were divided and analyzed according to the early, mid, and late phases. To eliminate potential human interference factors (e.g., abnormal behaviors such as coughing or talking), data collected 5 s before and after any abnormal behavior were regarded as contaminated data and excluded from subsequent analyses.

Indirect calorimetry formulas were used to convert existing energy metabolism indicator data into EE, with the formula as follows:

$$\text{EE }\left(\text{kcal}/\min\right)=\left(3.941\times {\text{VO}}_{2}\right)+\left(1.106\times {\text{VCO}}_{2}\right)$$

Additionally, based on the existing VO_2_ and RER data, the proportion of energy supply systems in different game phases was roughly estimated using the following formulas (Peronnet and Massicotte, 1991):

Fat oxidation rate (g/min): Fat(g/min) = 1.695×VO_2_×(1−RER).

Carbohydrate oxidation rate (g/min): CHO (g/min) = 4.585×VO_2_×RER−3.226×VO_2_

Fat energy supply (kcal/min): Fat(kcal/min) = Fat(g/min)×9.

Carbohydrate energy supply (kcal/min): CHO (kcal/min) = CHO (g/min)×4.

Proportion of fat aerobic energy supply: [Fat(kcal/min)/(Fat(kcal/min)+CHO(kcal/min))]×100%

Proportion of carbohydrate aerobic energy supply:

[CHO(kcal/min)/(Fat(kcal/min)+CHO(kcal/min))]×100%

Proportion of anaerobic energy supply: 1-Proportion of fat aerobic energy supply-Proportion of carbohydrate aerobic energy supply.

#### 2.5.2 Analysis of HRV indicators

Heart rate of subjects in different game phases was measured using the Polar H10 heart rate monitor, and HRV indicators were analyzed using Kubios software (University of Eastern Finland), including HR, R-R interval (RR), Root mean square of successive differences (RMSSD), High-frequency power (HF), and LF/HF ratio (LF/HF). To ensure data stability and result accuracy, only the HR data from the middle 10 min were retained during the resting state test, while the data from the first 5 min and last 5 min were excluded to eliminate interference from adaptation and recovery. During the game task phase, data were collected and analyzed according to the early-game, mid-game, and late-game phases.

Fast Fourier Transform (FFT) was used for frequency-domain analysis, with frequency bands defined per international standards: HF: 0.15–0.40 Hz; LF: 0.04–0.15 Hz. To reduce the impact of the extremely skewed distribution of HRV power units (ms^2^) on analysis, the mean values of HF and LF power were subjected to natural logarithm transformation (ln). This step was intended to enhance data distribution normality and the reliability of subsequent statistical analyses.

### 2.6 Statistical analysis

All statistical analyses were performed using SPSS 27.0 software. Data are presented as mean ± standard deviation for normally distributed variables, and median (interquartile range, IQR) for non-normally distributed variables. Accordingly, all demographic information was expressed as means, standard deviations and frequencies. Similarly, data regarding the proportional contribution of energy metabolism were presented as mean ± standard deviation. The physiology data was assessed for normality, where it was shown to be non-parametric. The data were then presented as medians and 25th and 75th percent interquartile ranges. The Wilcoxon signed-rank test was employed to examine differences in these indices across the three distinct game phases. In addition, a one-way repeated measures analysis of variance (ANOVA) was conducted to examine the proportional contribution of different energy metabolism pathways across the three game phases. Effect sizes were determined using partial eta squared η^2^). When a significant main effect was observed, *post hoc* pairwise comparisons were performed to identify specific differences between phases. Statistical significance was set at P < 0.05, with levels denoted as P < 0.001, P < 0.01, and P < 0.05 to ensure the accuracy of the results.

## 3 Results

### 3.1 Study on the proportion of different energy metabolism pathways across different game phases

The analysis of variance (ANOVA) revealed that there were distinct phase-dependent effects on the energy metabolism pathways (see Tables 3, 4). The anaerobic energy supply demonstrated an early-phase predominance, followed by significant attenuation in subsequent stages (F = 9.690, p = 0.001, η^2^ = 0.492). In contrast, the oxidation of carbohydrates exhibited a delayed activation pattern, characterised by a specific surge during the late phase (F = 8.109, p = 0.001, η^2^ = 0.323). Notably, the oxidation of fat followed a non-linear, inverted-U trajectory, displaying a transient mid-game peak before declining (F = 11.723, p < 0.001, η^2^ = 0.408).

**TABLE 3:** Proportion of energy metabolism pathways across different game phases.

Energy metabolism pathways	Early game (%)	Mid game (%)	Late game (%)
Anaerobic energy	13.3 ± 8.6	6.2 ± 8.4	7.6 ± 5.1
CHO oxidation	63.0 ± 7.4	63.6 ± 10.1	68.2 ± 11.7
Fat oxidation	23.7 ± 9.4	30.2 ± 10.1	24.2 ± 11.6

Data are presented as mean ± SD. CHO, carbohydrate oxidation.

**TABLE 4:** Post-hoc pairwise comparisons of energy metabolism pathway contributions.

Pathways	Comparison	P value	95% CI (lower, upper)
Anaerobic energy	Early vs. Mid game Early vs. Late game Mid vs. Late game	0.007 <0.001 0.803	0.030, 0.148 0.049, 0.142 −0.050, 0.063
CHO energy	Early vs. Mid game Early vs. Late game Mid vs. Late game	0.808 0.014 0.007	−0.053, 0.042 0.057, 0.446 0.081, 0.433
Fat energy	Early vs. Mid game Early vs. Late game Mid vs. Late game	0.004 0.012 0.001	−0.107, −0.024 0.026, 0.185 0.077, 0.266

CI: Confidence Interval. P-values indicate the statistical significance of pairwise comparisons.

### 3.2 Study on the characteristics of energy metabolism and HRV in E-sports athletes during the early game phase

**FIGURE 3:**
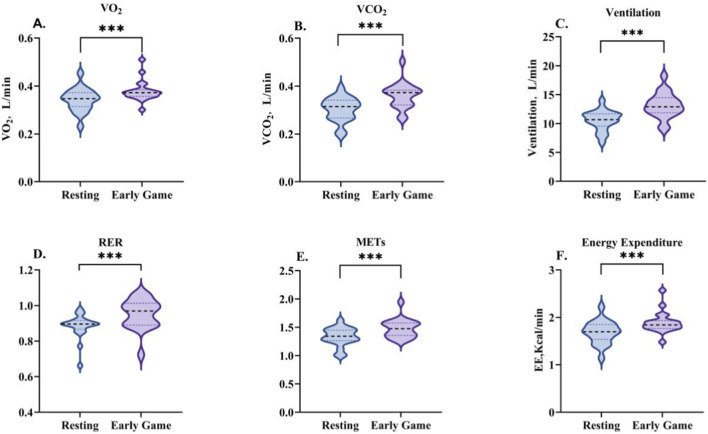
Changes in energy metabolism characteristics during the early-game phase. Data are presented as the median and interquartile range. VO_2_ **(A)**, VCO_2_ **(B)**, VE **(C)**, RER **(D)**, METs **(E)**, and EE **(F)**. *indicates a significant difference compared with the resting condition.

During the early phase of the game, there was a marked increase in energy metabolism indices in comparison to resting levels (see Table 5; Figure 3). Specifically, significant increases were observed in EE (Z = −3.733, P < 0.001), VO_2_ (Z = −3.733, P < 0.001), VCO_2_ (Z = −3.883, P < 0.001), VE (Z = −3.920, P < 0.001), RER (Z = −3.659, P < 0.001), and METs (Z = −3.733, P < 0.001).

**TABLE 5:** Comparative analysis of energy metabolism characteristics across different game phases.

Variables	Resting	Early game	Mid game	Late game
VO_2_ (L/min) VCO_2_ (L/min) VE (L/min)	0.34 (0.31–0.37) 0.31 (0.27–0.34) 10.67 (9.83–11.66)	0.37 (0.36–0.38)^***^ 0.37 (0.33–0.38)^***^ 12.88 (11.87–14.17)^***^	0.35 (0.34–0.37)^*††^ 0.34 (0.31–0.35)^***†††^ 12.44 (11.53–13.10)^***††^	0.36 (0.35–0.39) 0.34 (0.31–0.36) 12.47 (10.74–13.36)^*^
RER METs EE (Kcal/min)	0.89 (0.87–0.91) 1.34 (1.30–1.45) 1.70 (1.54–1.84)	0.97 (0.89–1.01)^***^ 1.48 (1.36–1.57)^***^ 1.84 (1.77–1.93)^***^	0.98 (0.88–0.97)^**†^ 1.39 (1.32–1.5)^*††^ 1.77 (1.68–1.85)^**†††^	0.95 (0.89–0.96)^†^ 1.45 (1.31–1.53) 1.81 (1.68–1.91)

Data are presented as median (interquartile range, IQR). VO_2_: oxygen uptake; VCO_2_: Carbon dioxide production; VE: minute ventilation; EE: energy expenditure; METs: Metabolic equivalents. *P < 0.05, **P < 0.01, ***P < 0.001 significantly different from Resting. †*P* < 0.05, ††*P* < 0.01, †††*P* < 0.001 significantly different from Early Game.

**FIGURE 4:**
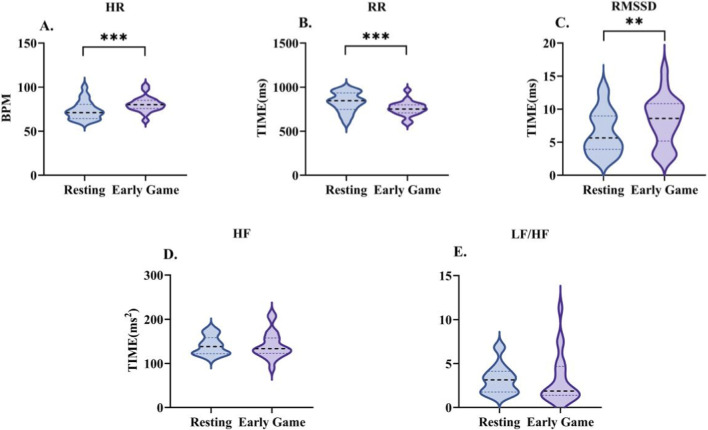
Changes in heart rate variability characteristics during the early-game phase. Data are presented as the median and interquartile range. HR **(A)**, R-R interval **(B)**, RMSSD **(C)**, HF **(D)**, and LF/HF **(E)**. *indicates a significant difference compared with the resting condition.

A comparison of the HRV (see Table 6; Figure 4) revealed a significant increase in heart rate (Z = −3.584, P < 0.001). Concurrently, the R-R interval demonstrated a significant decrease (Z = −3.584, P < 0.001). Furthermore, RMSSD demonstrated a substantial increase (Z = −3.118, P < 0.01). Despite the downward trends observed in both HF and the LF/HF ratio, these fluctuations did not attain statistical significance.

**TABLE 6:** Comparative analysis of HRV across different game phases.

Variables	Resting	Early game	Mid game	Late game
HR (bpm) RR (ms) RMSSD (ms)	71.0 (65.1–78.7) 846 (763–924) 5.65 (3.98–8.93)	80.1 (75.8–84.8)^***^ 751 (713–798)^***^ 8.60 (5.93–10.68)^**^	78.4 (73.5–83.3)^***^ 769 (723–818)^***^ 8.05 (5.58–10.63)^*^	77.8 (73.5–85.8) 780 (709–816) 9.60 (7.70–10.20)
HF(ms^2^) LF/HF	138 (123–154) 3.14 (1.81–4.00)	133 (123–156) 1.88 (1.44–4.26)	138 (128–156) 3.67 (2.14–4.68)^†^	131 (124–157) 2.48 (1.40–4.29)

Data are presented as median (interquartile range, IQR). HR: heart rate; RR: R-R interval; RMSSD: Root mean square of successive RR, interval differences; HF: High-Frequency Power; LF/HF: Ratio of low-frequency to high-frequency power. *P < 0.05, **P < 0.01, ***P < 0.001 significantly different from Resting. †*P* < 0.05, ††*P* < 0.01, †††*P* < 0.001 significantly different from Early Game.

### 3.3 Study on the characteristics of energy metabolism and HRV in E-sports athletes during the mid-game phase

**FIGURE 5:**
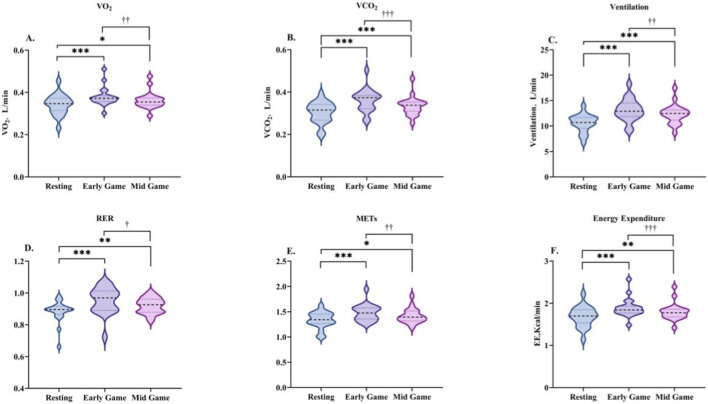
Changes in energy metabolism characteristics during the mid-game phase. Data are presented as the median and interquartile range. VO2 **(A)**, VCO_2_ **(B)**, VE **(C)**, RER **(D)**, METs **(E)**, and EE **(F)**. * indicates a significant difference compared with the resting condition, and † indicates a significant difference compared with the early-game phase.

During the mid-game phase, all energy metabolism indices exhibited a significant decline from the values observed in the early game phase, yet remained substantially elevated above resting levels (see Table 5; Figure 5). VO_2_ decreased from the early-game value (Z = −3.136, P < 0.01), yet remained above the resting value (Z = −2.501, P < 0.05). In a similar manner, the level of VCO_2_ was found to be lower than in the early game phase (Z = −3.453, P < 0.001), yet remained higher than at rest (Z = −3.808, P < 0.001). VE exhibited a similar trend, decreasing from the early game phase (Z = −2.949, P < 0.01) while remaining higher than the resting state (Z = −3.920, P < 0.001). RER decreased relative to the early game phase (Z = −2.427, P < 0.05), yet remained higher than resting values (Z = −2.725, P < 0.01). METs decreased from the commencement of the game (Z = −3.173, P < 0.01), yet remained above resting values (Z = −2.576, P < 0.05). Finally, EE decreased in comparison with the early game (Z = −3.360, P < 0.001), while remaining higher than resting values (Z = −2.912, P < 0.01).

**FIGURE 6:**
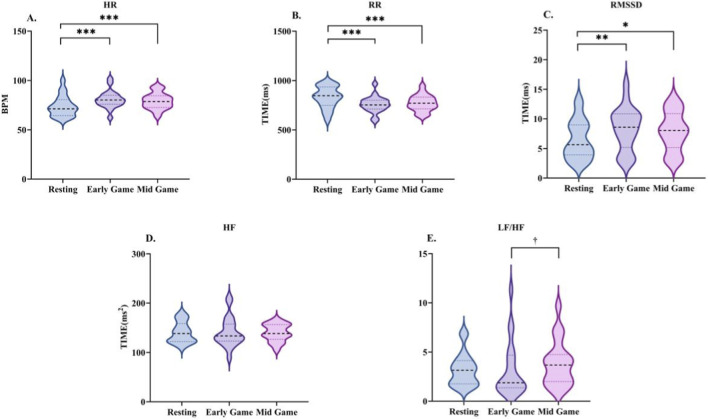
Changes in heart rate variability characteristics during the mid-game phase. Data are presented as the median and interquartile range. HR **(A)**, R-R interval **(B)**, RMSSD **(C)**, HF **(D)**, and LF/HF **(E)**. * indicates a significant difference compared with the resting condition, and † indicates a significant difference compared with the early-game phase.

During the mid-game phase, heart rate variability indices showed significant changes compared to the early game phase and the rest phase (see Table 6; Figure 6). HR decreased from the early game phase, but remained higher than during rest (Z = −3.435, P < 0.001). The R-R interval showed no change from the early game phase, but remained shorter than during rest (Z = −3.621, P < 0.001). RMSSD decreased from both the early game phase and the rest phase (Z = −2.523, P < 0.05). For the frequency-domain indices, there was no difference in HF from rest, while the LF/HF ratio increased from the early game phase (Z = −2.118, P < 0.05), showing an upward trend versus rest.

### 3.4 Study on the characteristics of energy metabolism and HRV in E-Sports athletes during the late game phase

**FIGURE 7:**
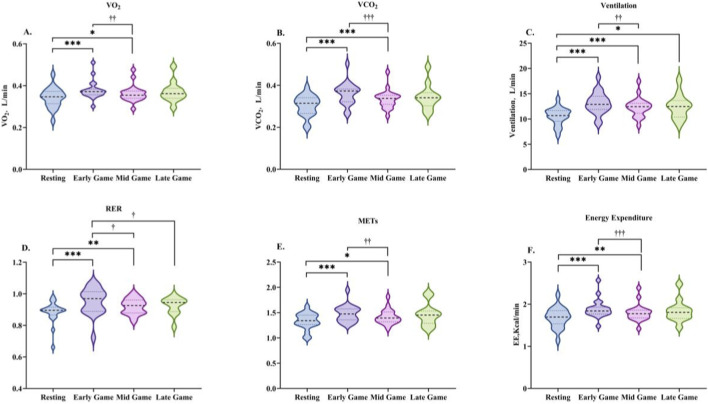
Changes in energy metabolism characteristics during the late-game phase. Data are presented as the median and interquartile range. VO2 **(A)**, VCO2 **(B)**, VE **(C)**, RER **(D)**, METs **(E)**, and EE **(F)**. * indicates a significant difference compared with the resting condition, and † indicates a significant difference compared with the early-game phase.

During the late game phase, the energy metabolism indices displayed a variety of trends in comparison to the other phases (see Table 5; Figure 7). VO_2_ and VCO_2_ were elevated above rest, but decreased from the early game phase (both non-significant). VE remained significantly higher than at rest (Z = −2.118, P < 0.05), with no difference compared to the early or mid-game phases. RER increased above rest, but decreased significantly from the early game phase (Z = −2.118, P < 0.05). METs and EE remained above rest values but below mid-game values, though these differences were not significant.

**FIGURE 8:**
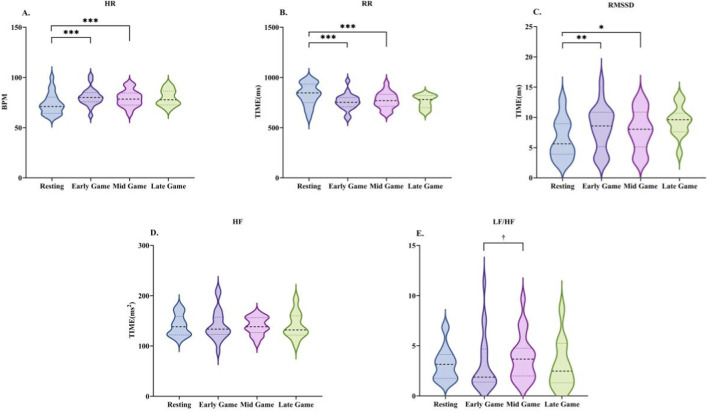
Changes in heart rate variability characteristics during the late-game phase. Data are presented as the median and interquartile range. HR **(A)**, R-R interval **(B)**, RMSSD **(C)**, HF **(D)**, and LF/HF **(E)**. * indicates a significant difference compared with the resting condition, and † indicates a significant difference compared with the early-game phase.

During the late-game phase, HRV indices exhibited sustained alterations (see Table 6; Figure 8). HR decreased compared to the early-game phase but remained elevated relative to the resting state (p > 0.05). The R-R interval was longer than in the early- and mid-game phases, but remained shorter than at rest (non-significant). RMSSD increased above rest, though this was not significant. For the frequency-domain indices, HF reached its lowest level across all phases and the LF/HF ratio decreased from the mid-game phase, though neither of these changes was statistically significant.

## 4 Discussion

The core characteristics of MOBA E-sports, namely, high strategic complexity, intensive operational demands, and phased cognitive load, pose sustained physiological challenges to the neuro-cognitive-metabolic systems. Growing evidence argues against classifying e-sports as sedentary activities, supported by studies demonstrating significant alterations in energy metabolism and heart rate variability during high-level competitive play. This suggests that e-sports should be regarded as having distinct physiological exercise relevance (Jenny et al., 2016; DiFrancisco-Donoghue et al., 2019).

### 4.1 Changes in energy metabolism and stress load characteristics of E-Sports athletes during the early-game phase

The early-game phase, defined by critical tactical preparation and acute cognitive demands, triggers rapid, high-intensity activation of both metabolic and stress-response systems. Far from reflecting a passive sedentary state, this phase exhibits a distinct physiological profile governed by the “cognitive stress-glycogenolysis” pathway. The observed predominance of carbohydrate oxidation (RER about 1.0) suggests that high cognitive load upregulates CNS-mediated glycogen phosphorylase activity to meet neuronal energy requirements (Mul et al., 2015). The subsequent increase in energy expenditure surpasses that observed during conventional mental activities (Fairclough and Houston, 2003) and amateur gameplay (Kocak, 2021). Collectively, these findings challenge the classification of e-sports as a sedentary activity. Notably, this magnitude of energy expenditure supports Hoshikawa and Yamamoto’s (1997) hypothesis that complex cognitive activities can independently modulate energy metabolism via autonomic pathways.

It is noteworthy that the magnitude of energy expenditure in these elite athletes corresponds with the distinction between elite and amateur physiological responses as outlined by Nicholson et al. (2024). The elevated EE observed here is likely indicative of the “cognitive automation” that has been developed through long-term training. In contrast to amateur players, elite athletes have conditioned responses to key information, which minimises cognitive resource waste and enables efficient allocation of metabolic substrates to high-level decision-making and motor precision. This finding indicates that the metabolic cost of e-sports is not a mere stress response, but rather a functional adaptation to high-performance cognitive processing.

Regarding autonomic regulation, the early-game phase was characterised by sympathetic-parasympathetic coactivation, as demonstrated by the concomitant elevation in heart rate and maintenance of vagal tone. This finding aligns with the “autonomic coordinated activation” theory described in sports psychology to describe competitive preparation stages (Laborde et al., 2017; Haupt et al., 2021). As proposed by Thayer et al. (2012), this bidirectional adjustment serves an adaptive function: sympathetic activation enhances cerebral perfusion and alertness, while retained parasympathetic modulation mitigates hyperarousal, thereby stabilising cognitive function for tactical execution (Thayer et al., 2012).

### 4.2 Changes in energy metabolism and stress load characteristics of E-sports athletes during the mid-game phase

The mid-game phase was characterised by frequent confrontations and high operational intensity, leading to a “metabolic plateau” (Lyons et al., 2011). This phase was marked by a significant elevation in energy expenditure above resting levels, despite a moderate decline from the early-game peak levels. This sustained elevation is indicative of the match’s persistent cognitive-motor demands. Consistent with the principles of substrate regulation under stable intensity outlined by Li (2013), carbohydrate oxidation served as the primary energy source to ensure cognitive efficiency. Notably, the observed upregulation of fat oxidation reflects an adaptive strategy. Specifically, the body flexibly modulates secondary substrates to preserve glycogen reserves while maintaining metabolic homeostasis. This shift underscores the metabolic flexibility of elite esports athletes in balancing immediate neural energy needs with endurance requirements.

With respect to autonomic function, this phase is characterised by a transition from acute reactivity to “strategic endurance”, a pattern that is consistent with the “parasympathetic drift” theory (Lehrer and Eddie, 2013). Specifically, renewed sympathetic dominance nderpins the enhanced vigilance essential for resource competition, while partial recovery of vagal tone acts as a protective buffer against physiological exhaustion. In contrast to the rapid-response physiological profile characteristic of the early-game phase, this functional adaptation enables the regulatory system to prioritise sustained cognitive performance over immediate reaction (Prinsloo et al., 2014; Shaffer et al., 2014).

A comparison of these results with previous findings reveals that physiological responses to esports vary substantially with game characteristics and player expertise. Studies on amateur players typically report the occurrence of “hemodynamic-metabolic decoupling”, a phenomenon in which cardiovascular stress (e.g., elevated heart rate) occurs in the absence of proportional metabolic activation (Haupt et al., 2021; Zimmer et al., 2022).

The primary physiological mechanism driving HR elevation during physical exertion is to increase cardiac output and deliver oxygen to tissues with high metabolic demand (Guyton and Hall, 2006; Duncker and Bache, 2008). Consequently, the coupled response observed in our elite cohort suggests that tachycardia is not solely a consequence of sympathoadrenal stress, but rather serves a functional homeostatic role in meeting actual metabolic costs (Carter and Goldstein, 2015). This interpretation is consistent with the findings of Nicholson et al., who also reported that high-level esports competition imposes measurable metabolic loads significantly above resting values (Nicholson et al., 2024). Consequently, while recreational gaming has been shown to induce stress-driven increases in heart rate independent of metabolism, elite competition appears to elicit a complex, coordinated activation of the cardiovascular and respiratory systems (Amann and Calbet, 2008).

### 4.3 Changes in energy metabolism and stress load characteristics of E-sports athletes during the late-game phase

The late-game phase, marked by the final consolidation of the win-loss trajectory and high-frequency motor actions, resulted in a modest rebound in energy metabolism. Despite the absence of statistically significant increases in VO_2_, VCO_2_, and energy expenditure relative to the mid-game period, the sustained predominance of carbohydrate oxidation (RER >0.9) highlights an adaptive prioritization of energy substrates. This substrate shift is hypothesised to facilitate the rapid ATP turnover required for tasks requiring high levels of operational accuracy and attentional focus, such as base defence and final team fights, where peak neuronal efficiency is necessary (Peronnet and Massicotte, 1991). This pattern is consistent with the findings of Nicholson et al. (2024), which indicates that the critical nature of the late game imposes a renewed load on energy metabolism to support high-stakes cognitive output. (Nicholson et al., 2024).

Regarding autonomic regulation, the late game exhibited a distinctive pattern of physiological divergence. While the decline in heart rate and rebound in RMSSD signaled the re-initiation of parasympathetic modulation, the continued suppression of HF power indicates a residual sympathetic constraint. This phenomenon, characterised by the mismatch between vagal reactivation and persistent sympathetic tone, aligns with the concept of ‘cognitive fatigue-related autonomic inhibition (Dantzer et al., 2008; Lim et al., 2010). The hypothesis is that the cumulative cognitive load induces a “regulatory latency”, where the body’s homeostatic drive to recover is partially inhibited by lingering neural fatigue. This interpretation is corroborated by Machado et al. (2022), who observed similar desynchronisation between HRV and heart rate in esports athletes (Machado et al., 2022), mirroring the compromised recovery profiles seen in high-stakes professions such as aviation and surgery.

Notably, the solo queue mode used in this study may have exerted a certain impact on the late-game stress load. Poulus et al. pointed out that factors such as teammate collaboration pressure and on-site audience atmosphere in team competitions significantly increase athletes’ psychological stress levels, leading to a higher LF/HF ratio in HRV and faster fatigue accumulation (Poulus et al., 2022). In the present study, the late-game LF/HF ratio was lower than that reported by Poulus et al. (2022) in their research on team-based competitions, suggesting that the stress load in solo-mode esports may be lower than that in actual team competitive scenarios. This limitation should be mitigated in future studies by incorporating team-based competitive paradigms into experimental designs.

### 4.4 Limitations

Although this study initially revealed significant changes in the energy metabolism and HRV profiles of esports athletes across different game phases, it still has certain limitations: (1) The study lacked female participants, potentially limiting the external generalizability of the findings; (2) It focused exclusively on League of Legends, without exploring how variations in game rhythms across other MOBA titles influence energy metabolism indices; (3) Solo queue matches were utilized as the measurement paradigm, rather than 5v5 team matches or in-person tournaments. Previous studies have shown that team-based competitions and on-site tournaments induce greater stress responses than solo matches (Poulus et al., 2022).

## 5 Conclusion

By analyzing the energy metabolism characteristics and HRV changes of E-sports athletes across different game phases, this study revealed the dynamic variations in physiological load and the underlying neural regulatory mechanisms during esports competition. The results showed that, during the early game phase, energy metabolism was dominated by carbohydrate oxidation, with anaerobic energy supply and fat oxidation contributing minimally. EE, VO_2_, and VCO_2_ all increased significantly; HR increased and the RMSSD value rose. These findings indicate that the activation of the sympathetic nervous system dominated the physiological responses in this phase. During the mid-game phase, energy metabolism tended to stabilize, and carbohydrate oxidation remained dominant. EE decreased, while HRV reflected the re-balancing of the sympathetic and parasympathetic nervous systems. This suggests that the neural regulatory system began to adapt to sustained cognitive load. During the late-game phase, EE exhibited a modest rebound yet manifested minimal overall variability. Alterations in heart rate and root mean square of successive differences reflected enhanced parasympathetic nervous system activity, which, however, did not revert to the resting state. This indicates that the body manifested a moderate degree of neural fatigue.

This study provides a physiological basis for the training and health management of E-sports athletes: Early-phase training should focus on enhancing energy mobilization capacity and tolerance to high-intensity cognitive loads; During the mid- and late-game phases, HRV monitoring may serve to evaluate neural regulatory status and fatigue accumulation, thereby facilitating the development of personalized training adaptation strategies; In the late-game phase, neural recovery intervention measures such as HRV biofeedback training can be incorporated to improve athletes’ fatigue resistance and competitive performance.

## Data Availability

The original contributions presented in the study are included in the article/Supplementary Material, further inquiries can be directed to the corresponding authors.
