# Supplementary figures and images for: Characteristics of energy metabolism and stress load in elite MOBA E-sports athletes
Source: Front Physiol. 2026 Feb 6;17:1716237. doi: 10.3389/fphys.2026.1716237 (PMC12920208; doi:10.3389/fphys.2026.1716237)

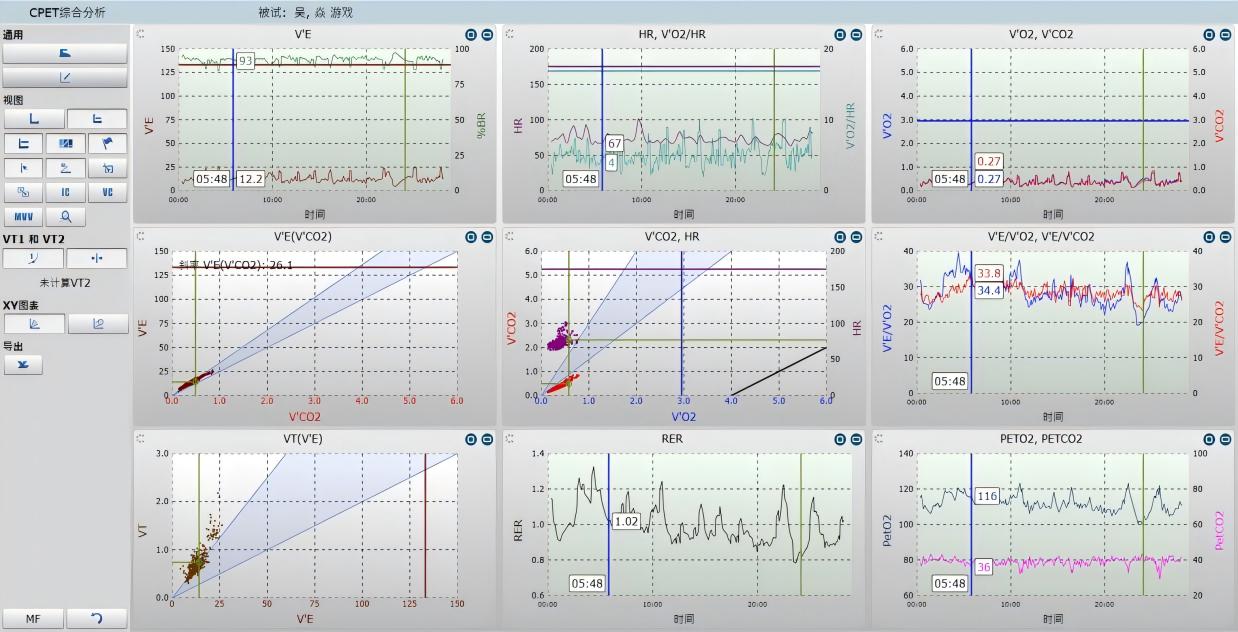

Supplement: Supplementary file 1 [file DataSheet1.zip › figure 1(A).jpg]

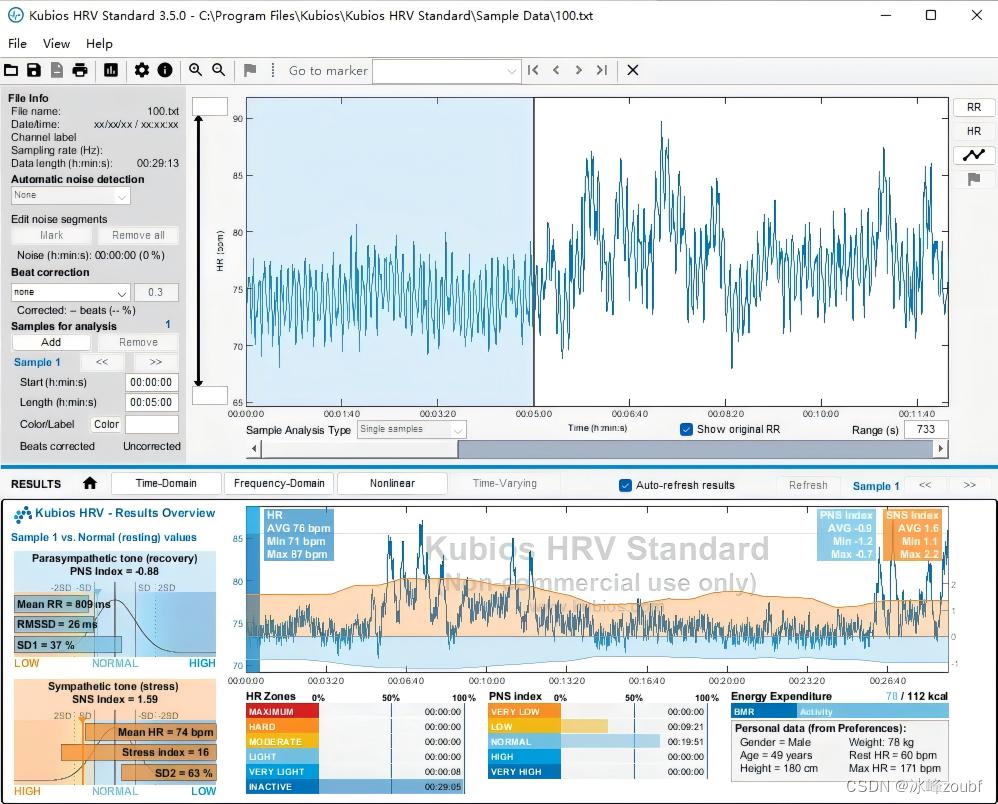

Supplement: Supplementary file 1 [file DataSheet1.zip › figure 1(B).jpg]

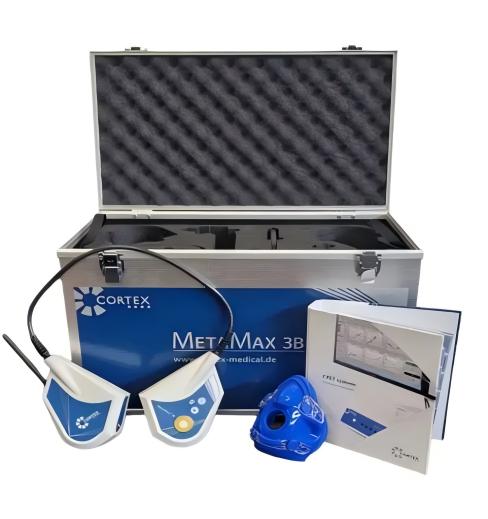

Supplement: Supplementary file 1 [file DataSheet1.zip › figure1(A).jpg]

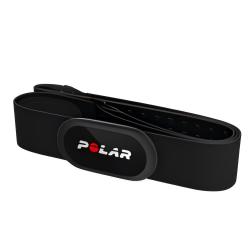

Supplement: Supplementary file 1 [file DataSheet1.zip › figure1(B).jpg]

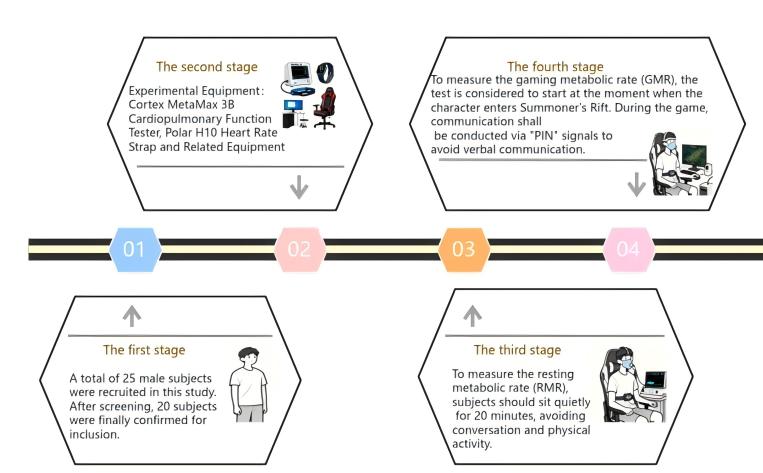

Supplement: Supplementary file 2 [file DataSheet2.zip › figure 2(A).jpg]

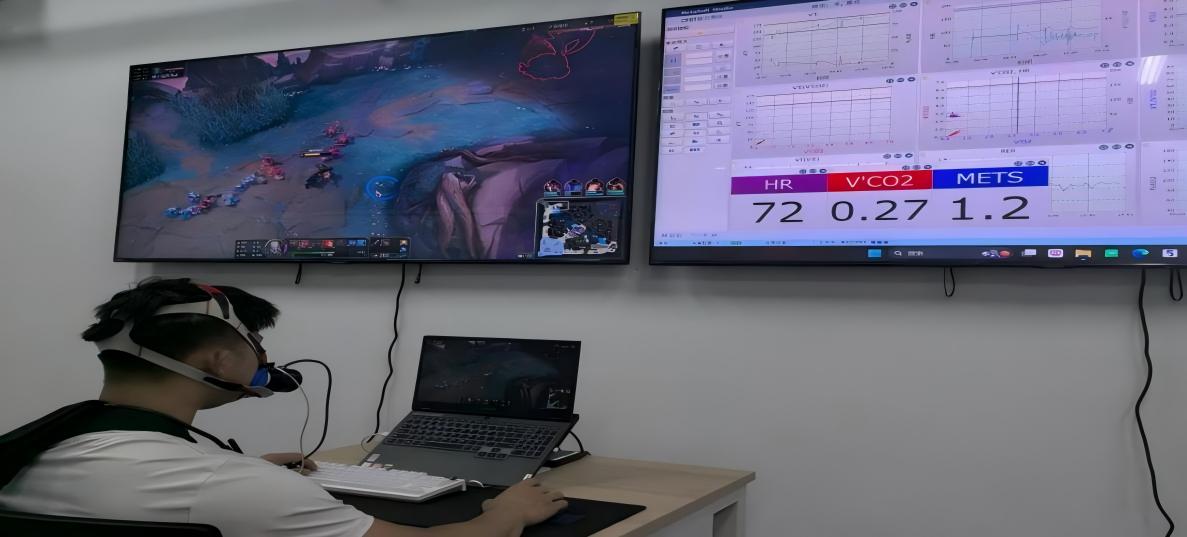

Supplement: Supplementary file 2 [file DataSheet2.zip › figure 2(B).jpg]
